# Efficient isolation of highly purified tonsil B lymphocytes using RosetteSep with allogeneic human red blood cells

**DOI:** 10.1186/1471-2172-10-30

**Published:** 2009-05-27

**Authors:** Jonathan Zuccolo, Tammy L Unruh, Julie P Deans

**Affiliations:** 1 Department of Biochemistry and Molecular Biology, Immunology Research Group, Institute of Infection, Immunity and Inflammation, Faculty of Medicine, University of Calgary, Calgary, Alberta, Canada

## Abstract

**Background:**

Human tonsils are a rich source of B lymphocytes exhibiting a variety of phenotypes and activation states. Existing methods of purification are time consuming or costly. The aim of the present study was to optimize conditions to isolate large numbers of highly purified primary B lymphocytes from tonsils in a short and cost-effective single step, using a commercially available reagent designed for purifying cells from whole blood (RosetteSep). This technique relies on the presence of the large excess of red blood cells in whole blood for the formation of immunorosettes, whereas single cell suspensions from tonsils contain relatively few red blood cells.

**Results:**

B cell enrichment from tonsils was achieved using RosetteSep with no modification to the whole blood procedure; however, the degree of purity depended on the extent of red blood cell contamination of the starting tonsil cell suspension. Addition of a 50-fold excess of allogeneic human red blood cells, but not sheep red blood cells, reproducibly resulted in high levels of purity. Depletion of mononuclear cells from the donor red blood cells eliminated potential contamination with allogeneic B cells.

**Conclusion:**

RosetteSep reagent can be used in combination with allogeneic human red blood cells to reproducibly isolate tonsil B lymphocytes to high levels of purity with no change in phenotype or loss of cells. This method provides considerable time and cost savings compared to other methods.

## Background

Human tonsils are a rich source of B lymphocytes exhibiting a variety of phenotypes and activation states. A well-established method for enriching B lymphocytes from tonsils involves the addition of 2-aminoethylisothiouronium bromide (AET) modified sheep red blood cells (RBCs) to deplete T lymphocytes by rosetting [1-3]. This is a relatively inefficient method, requiring advance acquisition and treatment of sheep RBCs, and several time-consuming steps on the day of cell separation. Additionally, while this method results in significant enrichment of B lymphocytes, there is often up to 8% contaminating T cells [2]. Magnetic cell separation techniques can yield highly purified populations of either negatively or positively selected B lymphocytes, but the high cost of reagents can be prohibitive when isolating very large numbers of cells.

In this study we describe a rapid and cost-effective approach for purifying large numbers of B lymphocytes from tonsils using a commercially available reagent, RosetteSep, which was designed for purifying cells from whole blood. The RosetteSep Human B cell Enrichment Cocktail consists of tetrameric antibody complexes with specificities for CD2, CD3, CD16, CD36, CD56, CD66b, and glycophorin-A. Addition of this antibody cocktail to whole blood results in crosslinking of unwanted cells with multiple RBCs to form immunorosettes that pellet when centrifuged over a lymphocyte separation density medium, while B lymphocytes collect at the interphase. This technique relies on the presence of the large excess of RBCs in whole blood for the formation of immunorosettes. Single cell suspensions from tonsils contain relatively few RBCs. Here, we show that by addition of human but not sheep RBCs to tonsil cell suspensions, RosetteSep can be used to rapidly obtain large numbers of highly purified B cells.

## Results

### Removal of lymphocytes from donor RBCs

**Figure 1:**
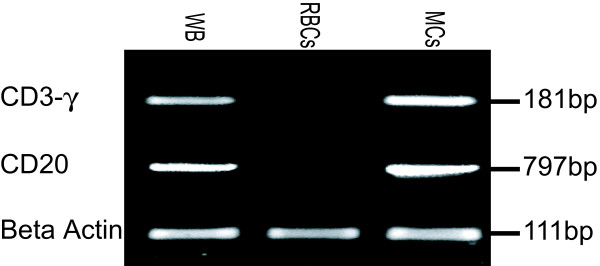
**Depletion of lymphocytes from donor blood**. To test for lymphocyte contamination of donor RBCs, cDNA was generated from whole blood (WB), purified RBCs and mononuclear cells (MC). CD20, CD3-γ and beta actin were amplified by PCR (n = 2).

We proposed to add allogeneic human RBCs to unfractionated tonsil cell suspensions in order to optimize the use of RosetteSep for B cell isolation from tonsils. First, it was necessary to consider potential contamination of the final product with B lymphocytes from the whole blood used to obtain RBCs. In healthy individuals white blood cells make up 0.08–0.22% of cells in the blood; B lymphocytes comprise 5–25% of this fraction, depending on the age of the donor [4,5]. Given the large excess of RBCs to be added to unfractionated tonsil cells, the use of human blood as a source of RBCs could result in significant contamination with non-tonsil B lymphocytes. In order to avoid contaminating tonsil B lymphocytes with allogeneic blood B lymphocytes during the purification, we first depleted mononuclear cells from the RBCs by centrifugation over lymphocyte separation medium. As expected, the mononuclear cells removed from whole blood were largely CD3 or CD19 positive as determined by flow cytometery (data not shown). A more sensitive measure, reverse-transcription PCR, was used to detect T and B cells in the RBC preparation before and after depletion (Figure 1). PCR products for both CD3 and CD20 were detected in samples of whole blood and the mononuclear cell fraction. The purified RBC preparation showed no CD3 or CD20 PCR product, indicating that it was free of lymphocyte contamination.

### Isolation of tonsil B lymphocytes using RosetteSep

**Figure 2:**
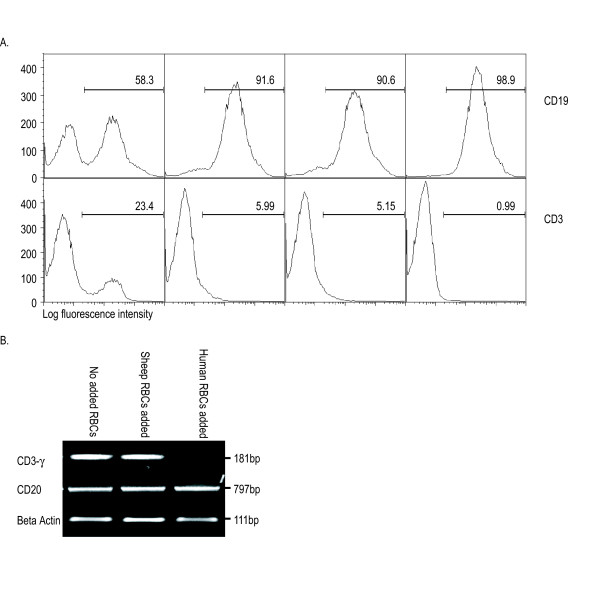
**Comparison of tonsil B lymphocytes isolated using RosetteSep with added human or sheep RBCs**. A. Unpurified tonsil cells, and B lymphocytes isolated using RosetteSep without added RBCs (n = 5), or with sheep RBCs (n = 2), or with human RBCs (n = 9), were analyzed by flow cytometry using anti-CD19 and anti-CD3, as indicated. Percentages of CD19-positive and CD3-positive cells in a representative experiment are shown. B. Reverse transcription PCR was used to further examine the purity of the tonsil B lymphocyte preparations. A PCR product for CD3-γ was amplified from cells purified with either no added RBCs or sheep RBCs (upper panel), but no PCR product was detected in cells purified using added human RBCs (n = 2). CD20 and beta actin were amplified to show that there were equal amounts of cDNA used for amplification.

Tonsil lymphocyte suspensions include 40–80% B lymphocytes, with T lymphocytes comprising the bulk of the remaining cells. Figure 2 shows a typical purification of B lymphocytes from tonsils using RosetteSep. In the unpurified tonsil cell suspension shown, there were 58.3% B cells and 23.4% T cells (Figure 2A). RosetteSep purification of B lymphocytes without the addition of RBCs resulted in approximately 91.6% purity with 6% T cell contamination (Figure 2A). Addition of human RBCs improved the purification to 98.9%, reducing the amount of T cell contamination to 1% (Figure 2A).

Sheep RBCs have been used for tonsil B lymphocyte isolation by RosetteSep [6]. However, we found that the addition of sheep RBCs provided no improvement in B lymphocyte purity over that obtained without added RBCs (Figure 2A). Additionally, we found that tonsil B cells purified with either sheep RBCs or no added RBCs gave a reproducible PCR product for CD3 after 35 cycles, whereas there was no evidence of CD3 in tonsil B lymphocytes purified with human RBCs (Figure 2B).

To assess cell loss during this procedure, we determined the percent of CD19+ lymphocytes in unfractionated tonsil cell suspensions using flow cytometry. After purification, the total number of recovered B cells was determined and expressed as a percentage of the estimated input number. It was found that this method of B lymphocyte purification reproducibly yielded greater than 99% recovery.

**Figure 3:**
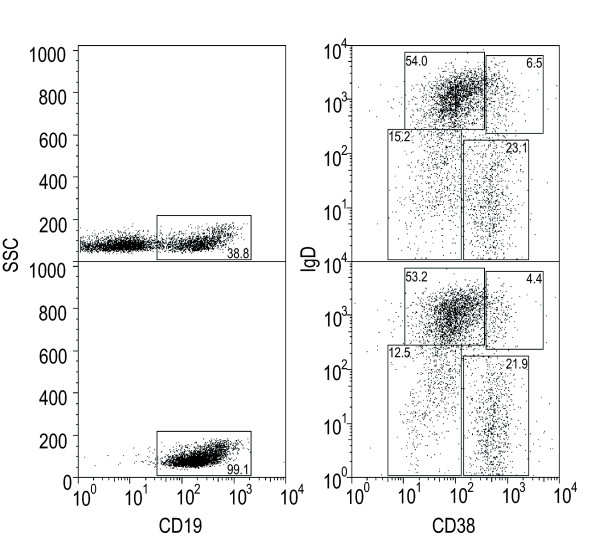
**Comparison of tonsil B lymphocyte phenotypes before and after purification**. Unpurified tonsil cells (upper panels) and purified B cells (lower panels) were stained with anti-CD19-TC, anti-IgD-PE and anti-CD38-FITC, and analyzed by flow cytometry. Unpurified tonsil lymphocytes were gated for CD19 expression before analyzing the IgD/CD38 profile (n = 5).

To further assess the utility of this method, the B lymphocyte phenotypes were examined before and after purification, using flow cytometry to detect CD19, CD38 and IgD. Unpurified tonsil lymphocytes were gated on CD19 expression before examining the CD38/IgD profile (Figure 3, upper panels). RosetteSep with the addition of human RBCs resulted in a 99% pure population of B-lymphocytes with a CD38/IgD profile similar to that of the corresponding tonsil cells, suggesting that all B lymphocyte populations are preserved during purification (Figure 3, lower panels).

## Discussion

We describe in this report the application of RosetteSep for isolation of tonsil B lymphocytes to very high purity. Significant enrichment can be obtained using RosetteSep without the addition of RBCs (Figure 2A), the degree of purity depending on the extent of RBC contamination of the starting tonsil cell suspension (data not shown). However, the addition of excess human RBCs was found to be essential for reproducibly obtaining B cell purity above 98%; sheep RBCs could not be substituted likely owing to species specificity of the anti-glycophorin-A antibody in the RosetteSep cocktail.

Potential contamination with blood B cells from the RBC donor was eliminated by depletion of mononuclear cells from the RBC preparation; RBCs could then be stored for several weeks without significant hemolysis. The RosetteSep method with added human RBCs represents a significant improvement over current methods (AET-treated sheep RBCs and selection with immunomagnetic beads). Compared to the AET-treated sheep RBCs method, there are significant time savings in the purification procedure as well as in the RBC preparation; the stability of stored human RBCs compared to AET-treated sheep RBCs is a further advantage. Purification using immunomagnetic beads is equally efficient, however, RosetteSep is less than 10% of the cost for purification of an equal number of B lymphocytes. Thus we recommend the use of this modified RosetteSep technique for obtaining any number of highly purified tonsil B lymphocytes.

## Conclusion

RosetteSep can be used in combination with allogeneic human RBCs to reproducibly isolate tonsil B lymphocytes to high levels of purity with no change in phenotype or loss of cells. This method provides considerable time and cost savings compared to other methods.

## Methods

### Antibodies and reagents

Phycoerythrin (PE) conjugated anti-IgD, anti-CD3 and isotype control, IgG1, were from Becton Dickinson (Mississauga, ON). Tri-color (TC) conjugated anti-CD19 was from Caltag (Burlingame, AL). FITC conjugated anti-CD38 was from Abcam (Cambridge, UK). RosetteSep human B lymphocyte enrichment cocktail was purchased from StemCell Technologies Inc. (Vancouver, BC). Sheep RBCs were purchased from Cedarlane Laboratories Ltd (Hornby, ON).

### Tonsils

Tonsils were obtained from the Alberta Children's Hospital with ethical approval. Cell suspensions were prepared by cutting the tissue into fine pieces using scissors or razor blades in RPMI 1640 (Gibco Laboratories, Grand Island, NY) supplemented with 10% fetal bovine serum (FBS) (Gibco). The tonsil tissue fragments were then forced through a nylon screen of 70 μm mesh size to disrupt the pieces into single cells. Cells were then washed twice with RPMI/FBS and counted.

### Preparation of human red blood cells

Human blood was collected from healthy donors into heparin vacutainers. Mononuclear cells were removed by diluting the whole blood with an equal volume of RPMI/FBS, layering over lymphocyte separation medium (MP Biomedicals LLC, Solon, OH), and then centrifugation at 1160 × g for 20 minutes with no brake. Cells at the interphase were aspirated and discarded, while the loosely pelleted RBCs were washed and returned to their original volume using Alsever's solution. Prepared RBCs were counted and used for up to one month. All steps were performed at room temperature.

### Purification of tonsil B lymphocytes

To purify B lymphocytes, 2 × 10^8 ^tonsil cells were mixed with a 50-fold excess (10^10^) of human or sheep RBCs. The cells were centrifuged at 350 × g for 5 minutes and then resuspended in 4 mL of RPMI/FBS. 200 μl of RosetteSep was then added; the sample was gently mixed by rocking and incubated for 20 minutes. The sample was then diluted with an equal volume of phosphate buffered saline (PBS) containing 2% FBS and 1 mM EDTA. Lymphocyte separation medium was carefully layered under the preparation and centrifuged for 20 minutes at 1160 × g with no brake. Cells at the interface were collected, washed and saved for analysis. All steps were performed at room temperature.

### Reverse transcription PCR

Up to 10^7 ^cells were used to prepare total RNA using the RNeasy mini kit (Qiagen, Valencia, CA). 5 μg of total RNA was reverse transcribed using Superscript III (Invitrogen, Carlsbad, CA) with oligo dT primers. Polymerase chain reaction (PCR) was performed in 25 μl volumes with TAQ PCR master mix (Qiagen, Valencia, CA) using 35 cycles of 1 min at 95°C, 1 min at 58°C, and 50 sec at 72°C. Primers for CD20: forward 5' GCA GCA ACG GAG AAA AAC TC 3' and reverse 5' GAA GAA GCG TGA CAA CAC AAG 3'. Primers for CD3: forward 5' CTG GGA AGT AAT GCC AAG GA 3' and reverse 5' TAG ACC CCA ACA GCA AGG AC 3'. Primers for Beta actin: forward 5' CAC TCT TCC AGC CTT CCT TCC 3' and reverse 5' GTG TTG GCG TAC AGG TCT TTG 3'. PCR products were resolved on 1% agarose Tris-Borate-EDTA (TBE) gels with ethidium bromide and photographed to observe relative signals of each amplified transcript.

### Flow cytometry

Cells were incubated with directly conjugated antibodies for 15 min at room temperature, washed and acquired on a flow cytometer (FACScan, Becton Dickinson). The data were analyzed using the FlowJo program (Tree Star Inc, San Carlo, CA).

## Authors' contributions

JZ carried out flow cytometry experiments, PCR experiments and drafted the manuscript. TU carried out flow cytometry experiments and contributed intellectual expertise to the flow cytometry data. JD assisted with the manuscript and experimental design. All authors read and approved the final manuscript.
